# Comprehensive Genomic Profiling of 274 Thymic Epithelial Tumors Unveils Oncogenic Pathways and Predictive Biomarkers

**DOI:** 10.1093/oncolo/oyac115

**Published:** 2022-06-24

**Authors:** Nicolas Girard, Clémence Basse, Alexa Schrock, Shakti Ramkissoon, Keith Killian, Jeffrey S Ross

**Affiliations:** Institut Curie, Institut du Thorax Curie Montsouris, Paris, France; Faculté de Médecine Simonbe Veil, UVSQ, Paris Saclay Campus, Versailles, France; Institut Curie, Institut du Thorax Curie Montsouris, Paris, France; Faculté de Médecine Simonbe Veil, UVSQ, Paris Saclay Campus, Versailles, France; Foundation Medicine, Cambridge, MA, USA; Foundation Medicine, Cambridge, MA, USA; Foundation Medicine, Cambridge, MA, USA; Foundation Medicine, Cambridge, MA, USA; SUNY Upstate Medical University, Syracuse, NY, USA

**Keywords:** thymoma, thymic carcinoma, targeted therapy, chemotherapy, immunotherapy

## Abstract

**Background:**

Thymic malignancies represent a heterogeneous group of rare thoracic cancers, which are classified according to the World Health Organization histopathologic classification, that distinguishes thymomas from thymic carcinomas. Data regarding the biology of those tumors are limited in the literature, and the vast majority have been obtained using surgical specimens from early-stage disease. Meanwhile, treatment of advanced, refractory thymic tumors currently relies on chemotherapy, with limited efficacy. Comprehensive genomic profiling (CGP) of advanced, refractory tumors would open some opportunities for innovative treatments.

**Patients and Methods:**

A total of 90 and 174 consecutive patients with thymoma or thymic carcinoma, respectively, for whom formalin-fixed, paraffin-embedded specimens from recurrent, refractory tumor were sequenced, were included. Sequencing was performed using hybridization-captured, adaptor ligation-based libraries to a mean coverage depth of >500× for up to 315 cancer-related genes plus 37 introns from 28 genes frequently rearranged in cancer.

**Results:**

Thymomas featured a low frequency of genomic alterations (average of 1.8/tumor), and low levels of TMB. The genomic alterations identified in more than 10% of cases were in the *CDKN2A/B* and *TP53* genes. Amplification in the *NTRK1* gene was found in an unresectable, stage III, type B3 thymoma. Thymic carcinomas featured a significantly higher frequency of alterations at 4.0/tumor (*P* < .0001). Clinically relevant genomic alterations were observed in the CDKN2A, KIT, and PTEN/PI3K/MTOR pathways. Elevated TMB in thymic carcinomas was uncommon with only 6% of cases featuring *≥*10 mutations/Mb.

**Conclusions:**

Our cohort is the largest available so far, reporting on CGP of thymic epithelial tumors in the setting of advanced disease. The identification of clinically relevant genomic alterations in the *KIT*, *PI3K*, *CDKN2A/B*, or *NTRK* genes provides a strong rationale for potential precision medicine approaches using targeted agents. A subset of thymic carcinomas show high tumor mutation burden, what may be a predictor of efficacy of immune checkpoint inhibitors.

## Teaser

This article reports on comprehensive genomic profiling of thymic epithelial tumors in the setting of advanced disease, identifying clinically-relevant genomic alterations in the KIT, PI3K, CDKN2A/B, or NTRK genes, which suggests potential precision medicine approaches using targeted agents. A subset of thymic carcinomas showing high tumor mutation burden may also be a predictor of efficacy of immune checkpoint inhibitors.

**Implications for Practice**

Comprehensive genomic profiling of a large cohort of advanced, refractory thymic malignancies indicates clinically relevant genomic alterations in the CDKN2A, KIT, and PTEN/PI3K/MTOR pathways, providing a strong rationale for potential precision medicine approaches. A subset of thymic carcinomas show high tumor mutation burden, what may be a predictor of efficacy of immune checkpoint inhibitors.

## Introduction

Thymic malignancies represent a heterogeneous group of rare thoracic cancers, which are classified according to the World Health Organization histopathologic classification, that distinguishes thymomas from thymic carcinomas^1^; thymomas are further subdivided into different types (so-called A, AB, B1, B2, and B3) based upon the relative proportion of the non-tumoral lymphocytic component, and the resemblance to normal thymic architecture.^1^ Thymic carcinomas are similar to their extra-thymic counterpart, the most frequent subtype being squamous cell carcinoma. From a clinical standpoint, thymomas are usually tumors with a tendency toward local progression rather than metastasis; by contrast, thymic carcinomas have a high risk of metastatic spread.^2,3^

The management of thymic epithelial tumors is a paradigm of cooperation between clinicians, surgeons, and pathologists from establishing the diagnosis to organizing the multimodal therapeutic strategy.^4^ Surgery is the mainstay of the curative-intent treatment of thymic malignancies, as complete resection represents the most significantly favorable prognostic factor on overall survival^5^; meanwhile, up to 30% to 50% of patients may present with advanced, recurrent, metastatic, or refractory tumors that require systemic treatment,^6^ which currently mostly consists of chemotherapy, providing with disease control in a majority of patients but limited response and progression-free survival rates, especially in the late-line setting.^7^

When the rarity of thymic epithelial tumors precludes large randomized trials to be conducted to assess innovative therapeutic agents, comprehensive genomic profiling (CGP) may represent an opportunity to fit in the unmet need of more effective treatment in refractory thymic epithelial tumors, through the identification of predictive biomarkers to develop precision medicine approaches.^8^ When previous approaches based on limited panels of gene had been disappointing,^9^ modern next-generation sequencing approaches allow the genotyping of high number of genomic alterations (GA).^10^ Moreover, these techniques allow the determination of tumor mutation burden (TMB) that helps the assessment of the benefit-risk ratio of immune checkpoint inhibitors, which may achieve prolonged responses but also unveil severe immune-related adverse events in patients with advanced thymic epithelial tumors.^11^

Here we took advantage of a multicenter, large series of thymic epithelial tumors for which comprehensive genomic profiling was conducted in a routine, clinical practice setting at the time refractory disease occurred, using a central, accredited next-generation sequencing platform for genotyping. Taken together, our results provide with a unique insight into molecular pathways driving thymic carcinogenesis, and a rationale for the use of targeted agents and immune checkpoint inhibitors.

## Methods

### Selection of Cases

This study was conducted based on a series of 127 299 clinical cases from patients with cancer, which were analyzed molecularly using CGP in a Clinical Laboratory Improvement Amendments-certified, College of American Pathologists (CAP)-accredited laboratory (Foundation Medicine, Cambridge, MA). From this series, 90 consecutive cases of thymoma, and 174 consecutive cases of thymic carcinomas were retrieved, which were analyzed from September 2012 to April 2017. Approval for this study, including a waiver of informed consent and an HIPAA waiver of authorization, was obtained from the Western Institutional Review Board (Protocol No. 20152817).

### Pathological Review

The pathologic diagnosis of each case was initially made at each tissue source site and was confirmed on routine hematoxylin and eosin stained slides, using the standard 2015 World Health Organization classification.^1^ Tissue of origin of thymoma and thymic carcinoma specimens were primarily the mediastinum (*n* = 26 (29%) and *n* = 75 (43%), respectively), the pleura or pericardium (*n* = 19 (21%) and *n* = 18 (10%), respectively), the lung (*n* = 20 (22%) and *n* = 20 (11%), respectively), the liver (*n* = 3 (3%) and *n* = 20 (11%), respectively), or the chest wall or diaphragm (*n* = 8 (9%) and *n* = 11 (6%), respectively). All samples forwarded for DNA extraction had to contain a minimum of 20% tumor nuclear area, compared with benign nuclear area. Staging was assessed according to the Masaoka-Koga system, that was standard at the time of specimen collection.^12^

### Comprehensive Genomic Profiling

In brief, ≥50 ng DNAs were extracted from 40 μM of tumor samples in formalin-fixed, paraffin-embedded tissue blocks. The samples were assayed by CGP using adaptor-ligation and hybrid capture performed for all coding exons of from 287 (version 1) to 315 (version 2) cancer-related genes plus select introns from 19 (version 1) to 28 (version 2) genes frequently rearranged in cancer. Sequencing of captured libraries was performed using the Illumina HiSeq technology to a mean exon coverage depth of >500×, and resultant sequences were analyzed for base substitutions, insertions, deletions, copy number alterations (focal amplifications and homozygous deletions), and select gene fusions, as previously described.^10^

### Bioinformatics Analysis

Clinically relevant genomic alterations (CRGA) were defined as alterations that are targetable by anti-cancer drugs available on the market or in registered clinical trials. Germline variants documented in the dbSNP database (dbSNP142; https://www.ncbi.nlm.nih.gov/snp/), with 2 or more counts in the ExAC database (https://gnomad.broadinstitute.org), or recurrent variants of unknown significance that were predicted by an internally developed algorithm^10^ to be germline were removed, with the exception of known driver germline events (eg, documented hereditary *BRCA1/2* and deleterious *TP53* mutations). Known confirmed somatic alterations deposited in the Catalog of Somatic Mutations in Cancer (COSMIC v62) were highlighted as biologically significant.^13^ All inactivating events (ie, truncations and deletions) in known tumor suppressor genes were also called as significant. To maximize mutation-detection accuracy (sensitivity and specificity) in impure clinical specimens, the test was previously optimized and validated to detect base substitutions at a ≥5% mutant allele frequency (MAF), indels with a ≥10% MAF with ≥99% accuracy, and fusions occurring within baited introns/exons with >99% sensitivity.^13^ Tumor mutational burden (TMB) was determined on 1.1 Mb of sequenced DNA for each case based on the number of somatic base substitution or indel alterations per Mb after filtering to remove known somatic and deleterious mutations.^14^ Microsatellite instability status (MSI) was calculated from 114 loci.

## Results

### Clinical and Pathological Characteristics

A total of 90 thymomas and 174 thymic carcinomas were analyzed; as per Foundation Medicine prerequisite for conducting CGP, all cases had to be clinically advanced and refractory to standard of care for recurrent disease. Patient’s characteristics are summarized in Table 1. For CGP, given the low incidence of GA, the 90 thymomas were organized into 2 groups based on previously reported studies.^2,15,16^ First group (*n* = 61) was made of thymomas type A (*n* = 2), AB (*n* = 17), B1 (*n* = 16), and B2 (*n* = 26), known to be low grade, given the rarity of cellular atypia^15-17^ and the lower stage at diagnosis,^1,17^ leading to a better survival^17^, second group (*n* = 29) was type B3 thymomas, which is considered as intermediate grade given presence of significant tumor cell atypia, and frequently invasiveness.^2,15-17^

**Table 1.** Clinical and genomic findings in thymomas and thymic carcinomas.

Histology	Thymoma		Thymic carcinoma						
Subtype	A/AB/B1/B2	B3	Adenocarcinoma	Basaloid	Lympho epithelioma-like	Neuroendocrine	Undifferentiated	Squamous	Sarcomatoid
Total	*n* = 61	*n* = 29	*n* = 7	*n* = 5	*n* = 5	*n* = 30	*n* = 54	*n* = 69	*n* = 4
Median age, yr	50	55	48	58	50	48	57	57	61
Male	28 (46%)	11 (38%)	4 (57%)	2 (40%)	4 (80%)	19 (63%)	41 (76%)	46 (66%)	2 (50%)
Female	33 (54%)	18 (62%)	3 (43%)	3 (60%)	1 (20%)	11 (37%)	13 (24%)	23 (34%)	2 (50%)
Mean number of GA/tumor	1.7	2.1	4.0	2.8	1.0	3.3	4.1	4.1	4.8
Mean number of CRGA/tumor	0.2	0.3	0.9	0.3	0	0.9	0.8	1.0	1.0
TMB ≥10 mut/Mb	2%	3%	14%	0%	0%	3%	5%	9%	0%
TMB ≥20 mut/Mb	0%	0%	0%	0%	0%	3%	5%	9%	0%

Abbreviations: GA, genomic alteration; CRGA, clinically relevant genomic alteration; TMB, tumor mutation burden.

Thymic carcinomas, given the limited amount of data in the literature regarding grading,^1^ were analyzed based on histologic subtypes: squamous cell carcinomas (*n* = 69, 40%), non-neuroendocrine undifferentiated carcinomas (*n* = 54, 31%), neuroendocrine carcinomas (*n* = 30, 17%), adenocarcinomas (*n* = 7, 4%), basaloid carcinomas (*n* = 5, 3%), lymphoepithelioma-like carcinomas (*n* = 5, 3%), and sarcomatoid carcinomas (*n* = 4, 2%).

### Comprehensive Genomic Profiling of Thymomas

**Figure 1.**
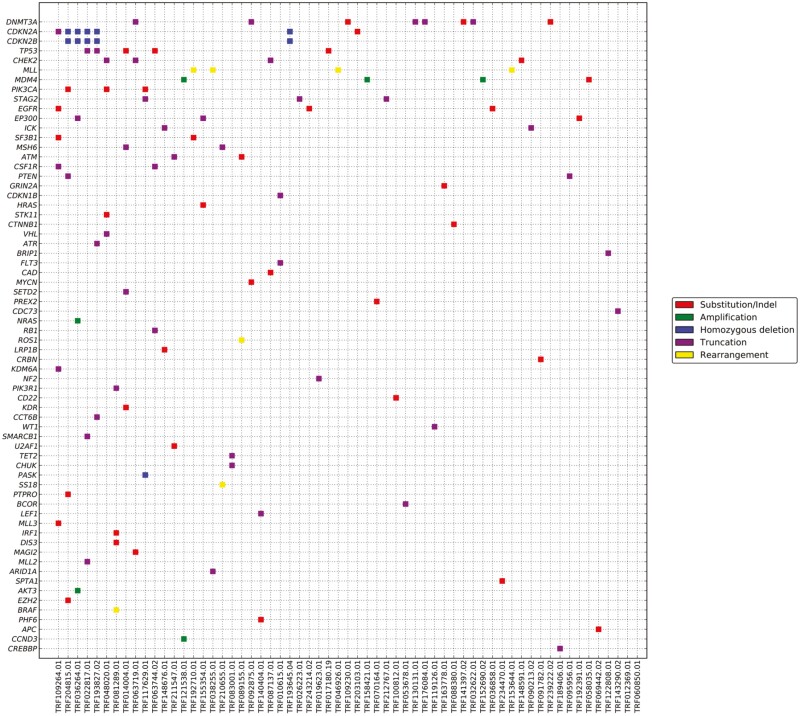

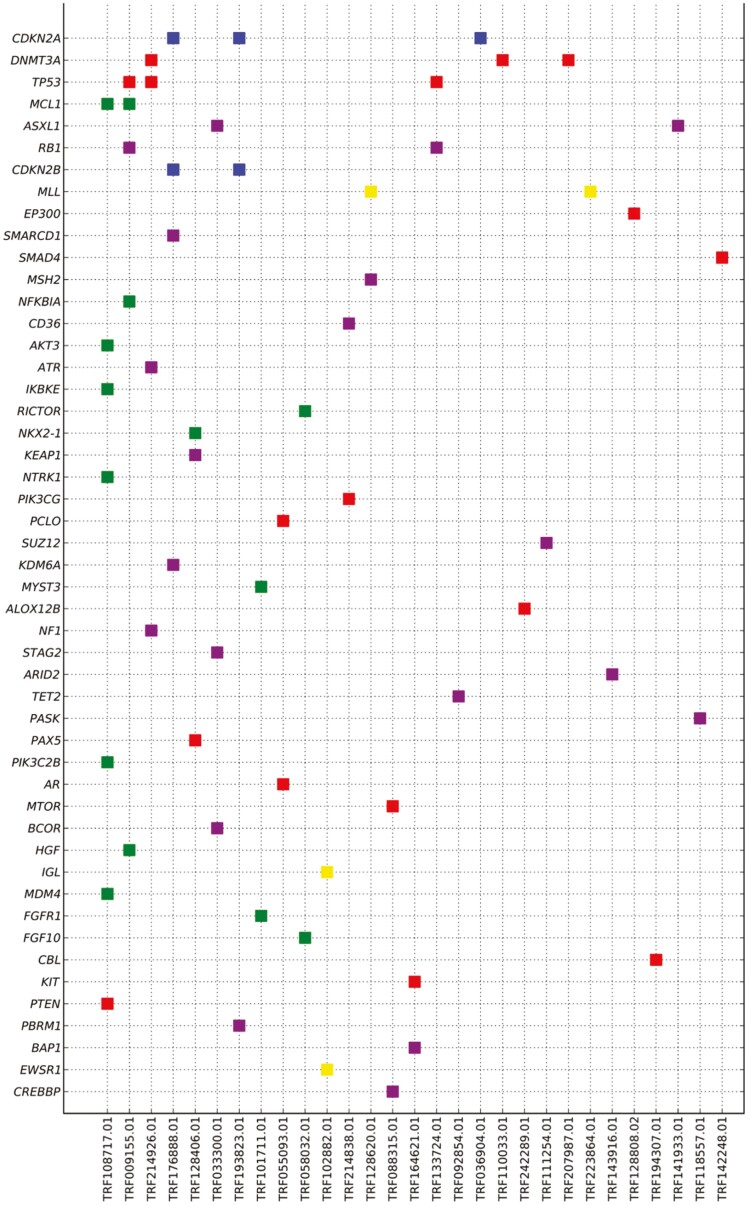
Somatically altered pathways in thymomas. OncoPrint diagrams depicting genomic co-alterations in selected genes in type A/AB/B1/B2 thymomas (**A**) and type B3 thymomas (**B**).

Overall, thymomas featured a low frequency of GA, CRGA and low levels of TMB. When comparing type A/AB/B1/B2 thymomas vs. type B3 thymomas, mean number of GA, CRGA were slightly more frequent in the latter group (P = N.S.; Table 1). The GA identified in more than 10% of cases were in the *CDKN2A/B* and *TP53* genes (Fig. 1A and 1B). CRGA were observed in less than 5% of cases, mostly in the MTOR pathway with alterations in the *PIK3CA*, *PTEN*, and *NF1* genes. A potentially targetable amplification in the *NTRK1* gene was found in an unresectable, stage III, type B3 thymoma. In another type B3 thymoma metastatic to the liver, a Y823D mutation in the *KIT* gene was identified. This alteration, however, is associated with resistance to available kinase inhibitors that target *KIT* activating mutations. All cases were MSI-stable. Only 2% of thymomas features a TMB ≥ 10 mutations/Mb; no thymoma had a TMB of *≥* 20 mutations/Mb.

### Comprehensive Genomic Profiling of Thymic Carcinomas

**Figure 2.**
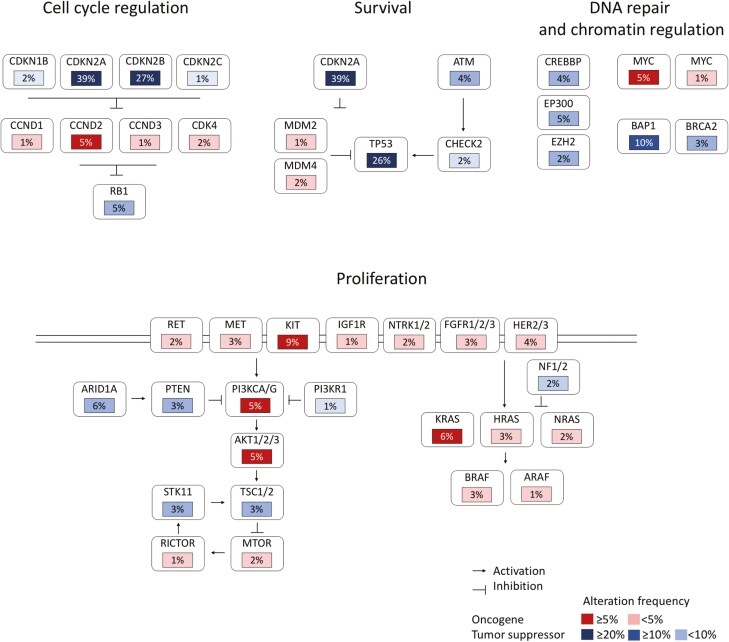

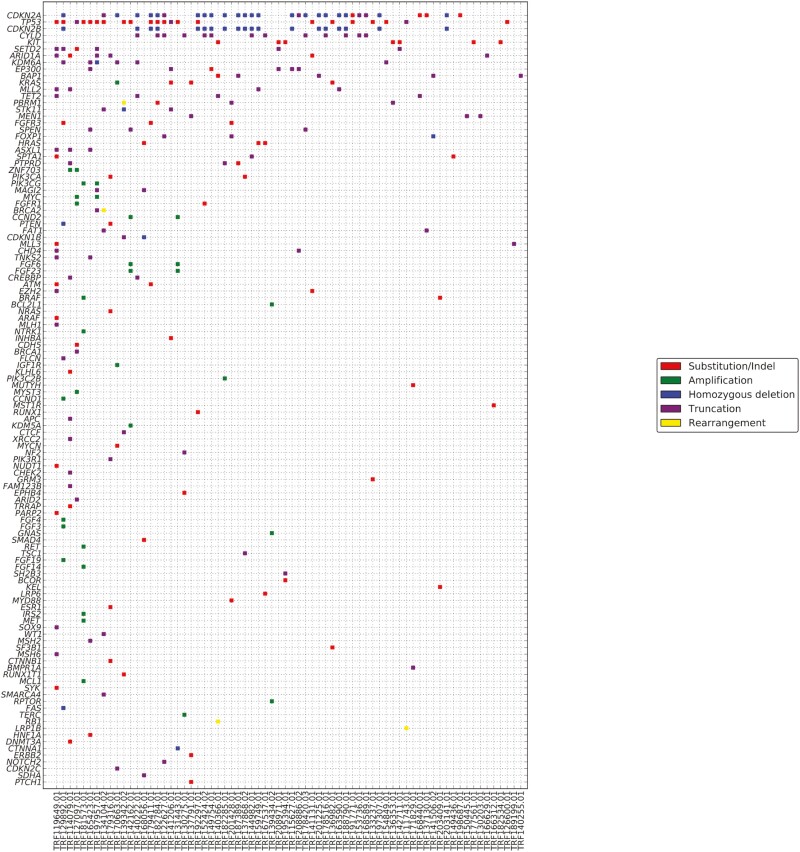

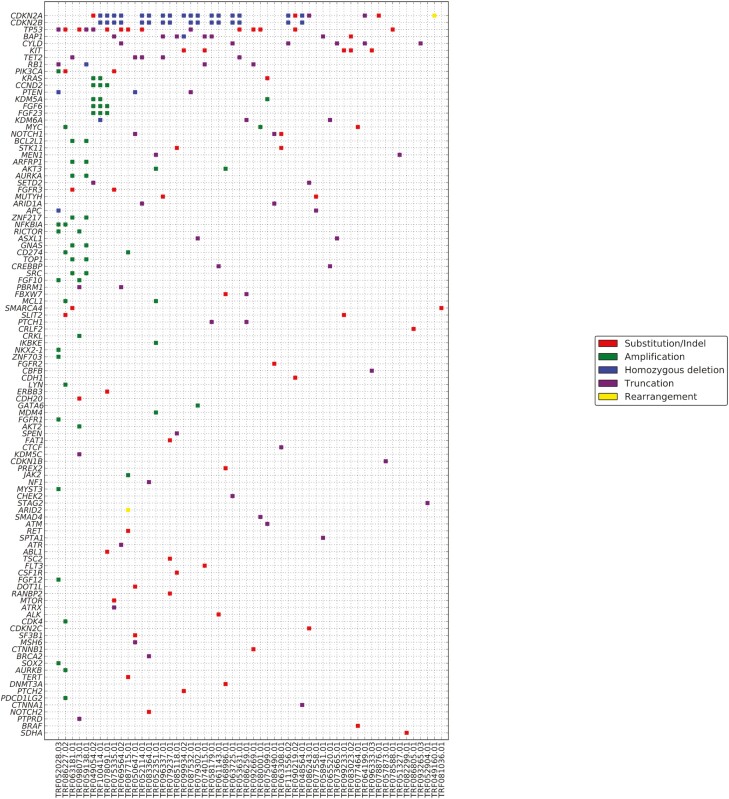

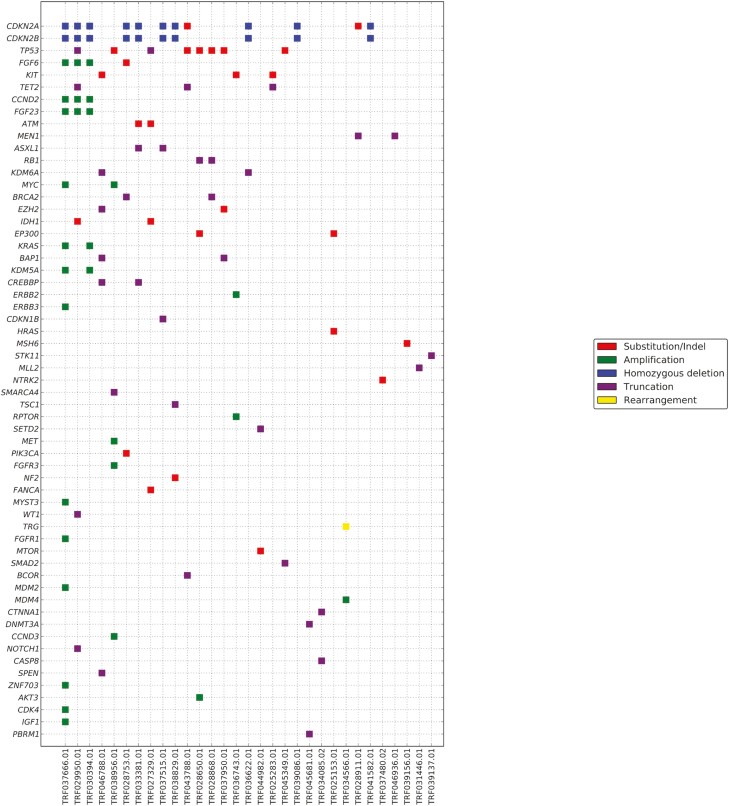
Somatically altered pathways in thymic carcinoma (**A**). Frequencies of alteration are expressed as a percentage of all cases, with background in red for activated genes and blue for inactivated genes (for colour figure refer to online version). OncoPrint diagrams depicting genomic co-alterations in selected genes in thymic squamous cell (**B**), undifferentiated (**C**), and neuroendocrine carcinomas (**D**).

The thymic carcinoma group, as a whole, featured a significantly higher frequency of GA at 4.0/tumor vs 1.8/tumor for thymomas (*P* < .0001), and significantly higher CRGA at 0.9/tumor versus 0.2/tumor for thymomas (*P* < .0001). GA were associated to deregulation of cell cycle, proliferation and survival, oxidative stress, and control of transcription pathways (Fig. 2A). Most frequent CRGA were observed in the CDKN2A, KIT, and PTEN/PI3K/MTOR pathways; other CRGA occurred in the *EZH2*, *MET*, *FGFR3*, and *ERBB2/3* genes. The median TMB was significantly higher in thymic carcinomas at 3.84 mut/Mb than in thymomas at 1.92 mut/Mb (*P* < .0001). Elevated TMB in thymic carcinomas was uncommon with only 6% of cases featuring *≥*10 mutations/Mb, and only 3% demonstrating *≥* 20 mutations/Mb (Table 1). All thymic carcinomas cases were MSI stable.

Among thymic carcinomas, GA tended to be more frequent in squamous, undifferentiated, and neuroendocrine carcinomas when compared with other subtypes (Table 1). Squamous cell and undifferentiated—non-neuroendocrine—carcinomas were the most frequent subtypes in our cohort, and featured *KIT* mutations in 9%-10% of cases (Fig. 2B and 2C). The pattern of GA and CRGA was globally similar in those 2 groups. The TMB was highest in squamous cell carcinomas, with 9% of cases featuring *≥*20 mut/MB. All cases of thymic neuroendocrine carcinomas included both low-grade (clinically malignant carcinoids) and high-grade tumors as well as small cell and large cell features and were confirmed by immunohistochemical staining for neuroendocrine markers. This group had nearly 1.0 CRGA/tumor with a variety of potential molecular targets including *KIT*, *MET*, *FGFR1*, and *TSC2* (Fig. 2D); only one case out of the 30 cases harbored a *TP53*-mutant/*RB1*-mutant profile.

No specific GA was observed among the less frequent histopathological subtypes of thymic carcinomas that all presented with various frequencies of *CDKN2A* alterations. All those cases were negative for evidence of association with Epstein-Barr virus infection.

## Discussion

In this study, we took advantage of a multicenter, large series of advanced, refractory thymic epithelial tumors for which comprehensive genomic profiling was conducted in a routine practice setting. Taken together, our results provide with a unique insight into molecular pathways activated in advanced thymomas and thymic carcinomas. Moreover, the identification of CRGA provides a strong rationale for potential precision medicine approaches using targeted agents. Ultimately, our results show that a subset of thymic carcinomas show high tumor mutation burden, what may be a predictor of efficacy of immune checkpoint inhibitors.

To our knowledge, our cohort is the largest reported so far, focusing on comprehensive genomic profiling of thymic epithelial tumors presenting with advanced disease; through the panel of genes that was analyzed, we were able to identify major deregulated pathways in refractory thymic carcinomas, including deregulation of cell cycle, proliferation and survival, oxidative stress, and control of transcription (Fig. 2A). Those deregulated pathways were previously identified at genomic characterization of limited cohorts of earlier stage, resected carcinomas.^8,18-20^ Resistance to apoptosis has been related to copy number gains of the anti-apoptotic molecule BCL2; in vivo exposure to a pan-BCL2 inhibitor leads to an inhibition of xenograft growth, via a mechanism involving the PI3K/AKT/mTOR pathway.^18^ Escape to growth suppressors has also been reported, with a deregulation of cell-cycle controlling molecules, including copy number loss of *CDKN2A/B*, hypermethylation of its promoter, accompanied by a lack of expression of its related protein p16INK4.^18^ Meanwhile, mutations in genes involved in histone modification, DNA methylation, and chromatin remodeling were also reported.^19^

GA are far less frequent in thymomas, although some alterations are shared with carcinomas, including *CDKN2A/B*, and *TP53* mutations. In The Cancer Genome Atlas study of 117 early-stage, resected thymic tumor specimens, molecular hallmarks of thymomas, which accounted for the majority of cases of this study, also included *GTF2I* mutations, specific for type A, and mutations in *HRAS* and *NRAS* mutations. These mutations are rare, and deciphering oncogenic mechanisms of carcinogenesis in thymomas remain challenging.

**Figure 3.**
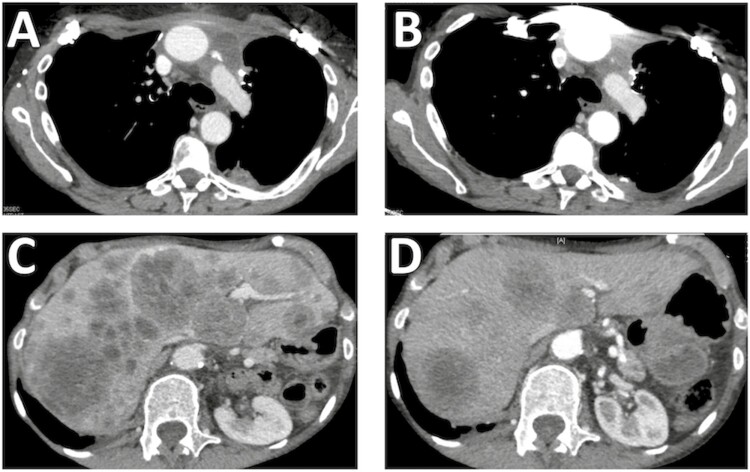
Response to everolimus in a 66-year-old women with refractory thymic carcinoma harboring a *PI3KCA* Glu545Lys mutation. The patient presented with stage IVB disease, with a mediastinal mass, pleural lesions associated with liver metastases. The patient previously received combination of Cisplatine, Adriamycin, and Cyclophophamide, then Carboplatin and Paclitaxel, followed by surgical resection of the mediastinal mass and mediastinal radiotherapy. The patient presented with recurrence treated with sunitinib, and subsequently everolimus. Objective response was observed after 3 months of treatment.

In the clinic, comprehensive genomic profiling of advanced thymic epithelial tumors primarily aims at identifying CRGA that may predict the efficacy of targeted agents, in the setting of refractory disease. *KIT*-mutant thymic carcinomas represent a small—9% of cases in our study—molecular subset of thymic carcinomas.^8^ The relevance of those mutations as a therapeutic target remains challenging as non-pretreated *KIT* mutants may not be uniformly sensitive to available KIT inhibitors, based on the clinical and/or the preclinical evidence in thymic carcinoma and/or other *KIT*-mutant tumors (https://www.mycancergenome.org/content/disease/thymic-carcinoma/). The *KIT* Y823D mutation we observed in a type B3 thymoma was previously not reported as being sensitive to KIT inhibitors. Ultimately, responses and possibly prolonged survival were reported with the off-label use KIT inhibitors—imatinib, sunitinib, or sorafenib—in patients with *KIT*-mutant thymic carcinoma, mostly in single-case observations^21^; phase II trials with sunitinib also identified such mutations among some long-term responders, but response observed in those studies was mostly attributed to the angioangiogenic effect through inhibition of vascular endothelial growth factor receptors.^22,23^ A second pathway of clinical relevance in thymic epithelial tumors is the PI3K pathway. Previously reported mutations mostly consist of missense mutations in *PIK3R2*, which encodes a regulatory subunit of PI3K.^24^ Activation of PI3K may also be related to the overexpression of a large microRNA cluster on chromosome 19 in type A and AB thymomas.^25^ PI3K targeting may then be an effective strategy in the treatment of thymic malignancies; a phase II study with buparlisib is currently ongoing (clinicaltrials.gov ID NCT02220855) (Fig. 3). Several phase I/II studies of mTOR inhibitors were reported in advanced thymic epithelial tumors, reporting on high disease control rates.^26,27^ With regards to other CRGA, one new finding of our study is the identification of potentially targetable amplification in the *NTRK1* gene in an unresectable, stage III, type B3 thymoma.^28^ Taken together, our findings support the development of trials or prospective cohorts combining routine use of comprehensive genomic profiling of refractory thymic epithelial tumors to identify CRGA, which are actually diverse and rare at the individual level, together with treatment with targeted agents; such umbrella strategies are needed to actually assess the value of such precision medicine approach, that demonstrated a survival benefit in other solid cancers, such as lung cancers.^29^

The use of immune checkpoint inhibitors is challenging in thymic epithelial tumors; immunotherapy has actually been contra-indicated in thymomas, as one third of patients present with autoimmune disorders, the most frequent being myasthenia gravis.^4^ Auto-immune disorders are usually not observed in thymic carcinomas, but exposure to targeted agents such as sunitinib^22^ or anti-PD-1 antibodies,^11^ was reported to unveil severe immune-related toxicities, including myositis, myocarditis, or diabetes. Pembrolizumab may still represent an effective treatment in the advanced setting; a phase II trial conducted in 41 patients reported a 23% objective response rate, with a median duration of response of 22 months.^11^ PD-L1 expression was not a predictor of response or survival in this study, what may be related to the fact that PD-1-PD-L1 interaction plays a physiological role in the modulation of lymphocytes selection in the thymus.^30^ Tumor mutation burden represents a predictive biomarker of efficacy of immunotherapy and may represent a valuable tool in the clinic to better select patients with thymic epithelial tumors for anti-PD-1 antibodies.^31^ Our results indicate that only 5%-9% of thymic carcinomas harbor a tumor mutation burden of 10 mutations/Mb, a cutoff previously reported as clinically relevant in lung cancer using the same technology.^31^ Further studies are needed to validate such correlation in thymic epithelial tumors.

To conclude, comprehensive genomic profiling may be of high value for the management of advanced, refractory thymic epithelial tumors. CRGA, even if rare in those tumors, may predict the efficacy of innovative targeted agents. Meanwhile, assessment of tumor mutation burden may help the decision-making of clinicians when discussing treatment with immune checkpoint inhibitors, what needs to be further validated. Ultimately, while a better understanding of the carcinogenesis of thymic epithelial tumors is still warranted, implementation of comprehensive genomic profiling suits with the unmet need of personalized approaches for those rare thoracic tumors.

## Data Availability

The data underlying this article will be shared on reasonable request to the corresponding author.

## Previous Presentation

This work was presented as an oral abstract at the European Society for Medical Oncology Annual Meeting 2017, Madrid, Spain.
